# Elevated Lipoprotein(a) Is Common in People With Familial Hypercholesterolemia and Negative Genetic Screening

**DOI:** 10.1016/j.jacadv.2025.101648

**Published:** 2025-03-17

**Authors:** Francis Gainsborough, Paul Welsh, Naveed Sattar, Maurizio Panarelli

**Affiliations:** a Department of Clinical Biochemistry, Glasgow Royal Infirmary, NHS Greater Glasgow and Clyde, Glasgow, United Kingdom; b University of Glasgow, School of Cardiovascular and Metabolic Health, Glasgow, United Kingdom

**Keywords:** genetics, lipids, routine care, screening

**What is the clinical question being addressed?**

Can we determine if elevated Lp(a) levels are more common in patients with a clinical presentation of familial hypercholesterolemia in whom no mutation was identified?

**What is the main finding?**

Results show high rates (>50%) of an alternative, genetic lipoprotein-mediated contributor to ASCVD, suggesting routine Lp(a) measurement in all such patients globally.

Lipoprotein (a) [Lp(a)] is a largely genetically determined cardiovascular risk marker that is strongly and monotonically associated with composite atherosclerotic cardiovascular disease (ASCVD) independently of other traditional cardiovascular risk factors.^1^ Furthermore, there is strong genetic evidence for its causal relationship with ASCVD and very high levels of Lp(a) (>200 mg/dL) may represent a comparable lifetime risk of coronary heart disease to genetically confirmed familial hypercholesterolemia (FH) while being up to twice as prevalent.^2^ Despite this, understanding of its clinical relevance in cardiovascular risk assessment is limited in many medical circles.

Given the possibility that elevated Lp(a) levels can inflate measured low-density lipoprotein-cholesterol (LDL-C) levels, there is a risk of misdiagnosing FH when LDL-C levels are evaluated without considering Lp(a) measurements. Accurate differentiation between phenotypic FH and genetically confirmed FH, in the context of Lp(a) levels, is crucial given also that the Lp(a) component of measured LDL-C is not lowered by conventional lipid-lowering agents. In this article, pseudo-FH refers to a presentation of possible FH with negative FH genetics. Based on a 2024 focused update of National Lipid Association 2019 guidelines, Lp(a) >50 mg/dL (>125 nmol/L) is considered to be high risk and <30 mg/dL (<75 nmol/L) low risk, with an intermediate risk category between these 2 values.^3^ When considering the general population, between 16% to 24% of people may have Lp(a) >50 mg/dL and around 35% >30 mg/dL.^1^^,^^4^

The aim of this work was to determine Lp(a) levels in a cohort of patients with pseudo-FH.

## Methods

Data were gathered as part of audit of the cardiovascular risk prevention clinic at the Glasgow Royal Infirmary, Scotland. Retrospective data were assimilated from clinic letters, genetic, and biochemistry results for 2023. Patients included were those who had been referred to the clinic, for whom there was a suspicion of FH based on the Simon Broome criteria, who had an Lp(a) measured and for whom no FH mutation was identified. Suspicion of FH was defined as secondary-care consultant authorizing FH genetic studies. Lp(a) was measured by immunoturbidimetric assay by Allinity c. Calibrators were standardized to an in-house Abbot reference material with values reported in mg/dL. FH genetic analysis reported mutations associated with low density lipoprotein receptor, proprotein convertase subtilisin/kexin type 9, apolipoprotein B, low-density lipoprotein receptor adapter protein 1, and apolipoprotein E. Caldicott approval was obtained. Comparison to the UK Biobank cohort was performed using the full cohort of those with available recruitment visit measures of Lp(a), total cholesterol, and direct LDL-C. Lp(a) was measured as previous described^1^ and units were converted from nmol/L to mg/dL by dividing by 2.5. P.W. and F.G. had full access to all the data in the study and take responsibility for its integrity and the data analysis.

## Results

In 2023, 81 patients met study inclusion criteria. This number was derived from 158 clinic referrals of whom 94 had suspected FH with 89 of these also having an Lp(a) result. Eighty-one of these had negative FH genetics. The mean age was 55.8 ± 10.7 years, and 59 (72.8%) were female (Table 1). At the time of referral, mean total cholesterol in the 81 patients with negative FH genetics was 310.1 ± 45.5 mg/dL) and LDL-C was 213.1 ± 44.6 mg/dL). Median Lp(a) was 30.8 mg/dL (IQR: 8.8-94.1 mg/dL) with Lp(a) (n = 34 [42%] >50 mg/dL). Ethnicity data were not recorded; however, this was a predominantly (>90%) White cohort. Lp(a) was elevated in the pseudo-FH cohort relative to UK Biobank (+188%) to a greater extent than total cholesterol (+40%) and LDL-C (+54%) (Table 1).

**Table 1:** Comparison of Lipid Levels in the Pseudo-FH Cohort to UK Biobank

	Pseudo-FH Cohort	UK Biobank	*P* Value	Age- and Sex-Adjusted Effect of Pseudo-FH^a^
Age	55.8 ± 10.7	56.5 ± 8.1	0.40	-
Female	59 (72.8%)	245,930 (54.2%)	<0.001	-
Total cholesterol (mg/dL)	310.1 ± 45.5	220.1 ± 44.3	<0.001	OR: +40% (95% CI: +35 to +45%)
LDL cholesterol (mg/dL)	213.1 ± 44.6	137.5 ± 33.6	<0.001	OR: +54% (95% CI: +45 to +64%)
Lp(a) (md/dL)	30.8 (8.8-94.1)	7.9 (3.0-30.0)	<0.001	OR: +188% (95% CI: +144 to +286%)
Lp(a) category			<0.001	
<30 mg/dL	39 (48.1%)	340,317 (75.0%)		Ref
30-50 mg/dL	8 (9.9%)	38,630 (8.5%)		OR: 1.80 (95% CI: 0.84-3.86)
≥50 mg/dL	34 (42%)	74,731 (16.5%)		OR: 3.89 (95% CI: 2.45-6.17)

Values are mean ± SD, n (%), or median (IQR) unless otherwise indicated.

FH = familial hypercholesterolemia; LDL = low-density lipoprotein; Lp(a) = lipoprotein (a).

a Effect estimates represent the ratio of geometric means from exponentiated beta-coefficients obtained using linear regression on log-transformed data (for continuous Lp(a), total cholesterol, and LDL cholesterol) or ORs for Lp(a) categories.

## Discussion

In a cohort of patients with so-called pseudo-FH, we demonstrate a high prevalence of elevated Lp(a) levels. These results demonstrate high rates of an alternative, genetic lipoprotein mediated, and at least partial cause, of increased ASCVD risk in this clinically challenging group of patients. That 52% of such patients had a Lp(a) >30 mg/dL and 42% had levels >50 mg/dL shows that routine Lp(a) measurement should be considered in all patients being screening for FH.

The data suggest a 188% increase in Lp(a) and 3.9-fold increased odds of elevated Lp(a) when compared to UK Biobank. The Lp(a) values reported herein are also elevated compared to the general patient population of the United States.^4^ Given that the risk-concentration relationship appears positively linear, these patients are at significant risk of ASCVD when compared to the general population. This is of clinical pertinence given the low rates of Lp(a) testing in many clinical settings.

In these findings, higher Lp(a) levels are likely to contribute to polygenic FH. This should be considered when formulating an individual’s cardiovascular risk and planning appropriate cascade testing. While European Atherosclerosis Society guidance calls for Lp(a) measurements at least once in all patients, such testing has been sparsely adopted in many settings due to cost, lack of knowledge, and no definitive treatments for elevated Lp(a).^5^ Thus, we need to better define groups who would value from such measurements, at least until major outcome trials report. This work suggests that prioritizing patients with pseudo-FH may be an appropriate place to begin wider testing.

In conclusion, patients with pseudo-FH show increased prevalence of high Lp(a) compared to the general population. That 1 in 2 patients with such a history have at least moderately elevated Lp(a) levels (>30 mg/dL) means that many patients can be better incentivized to consider lipid-lowering therapy. Such findings further recommend the need to include routine Lp(a) testing in all patients suspected of having FH.

## Funding support and author disclosures

Dr Sattar has consulted for and/or has received speaker honoraria from Abbott Laboratories, AbbVie, Amgen, AstraZeneca, Boehringer Ingelheim, Carmot Therapeutics, Eli Lilly, GlaxoSmithKline, Hanmi Pharmaceuticals, Janssen, Menarini-Ricerche, Metsera, Novartis, Novo Nordisk, Pfizer, Roche Diagnostics, and Sanofi; and has received grant support paid to his University from AstraZeneca, Boehringer Ingelheim, Novartis, and Roche Diagnostics outside the submitted work. Dr Welsh has received grant support from AstraZeneca, Roche diagnostics, Boehringer Ingelheim, and Novartis outside the submitted work and has received honoraria from Novo Nordisk and Raisio outside the submitted work. All other authors have reported that they have no relationships relevant to the contents of this paper to disclose.
